# Determinants of Survival and Post-Progression Outcomes by Sorafenib–Regorafenib Sequencing for Unresectable Hepatocellular Carcinoma

**DOI:** 10.3390/cancers14082014

**Published:** 2022-04-15

**Authors:** I-Cheng Lee, Yee Chao, Pei-Chang Lee, San-Chi Chen, Chen-Ta Chi, Chi-Jung Wu, Kuo-Cheng Wu, Ming-Chih Hou, Yi-Hsiang Huang

**Affiliations:** 1 Division of Gastroenterology and Hepatology, Department of Medicine, Taipei Veterans General Hospital, Taipei 11217, Taiwan; iclee@vghtpe.gov.tw (I.-C.L.); pclee11@vghtpe.gov.tw (P.-C.L.); ctchi2@vghtpe.gov.tw (C.-T.C.); cjwu6@vghtpe.gov.tw (C.-J.W.); kcwu3@vghtpe.gov.tw (K.-C.W.); mchou@vghtpe.gov.tw (M.-C.H.); 2 School of Medicine, National Yang Ming Chiao Tung University, Taipei 11221, Taiwan; 3 Cancer Center, Taipei Veterans General Hospital, Taipei 11217, Taiwan; ychao@vghtpe.gov.tw (Y.C.); scchen16@vghtpe.gov.tw (S.-C.C.); 4 Institute of Clinical Medicine, National Yang Ming Chiao Tung University, Taipei 11221, Taiwan

**Keywords:** hepatocellular carcinoma, sorafenib, regorafenib, progression-free survival, overall survival, post-progression survival

## Abstract

**Simple Summary:**

The optimal subsequent treatment and the determinants of survival after sorafenib–regorafenib failure in patients with hepatocellular carcinoma (HCC) remain unclear. The aim of this study was to delineate the determinants of response and survival after regorafenib and evaluate the post-progression outcomes in the era of multiple-line sequential systemic therapy. We retrospectively enrolled 108 patients with unresectable HCC receiving regorafenib after sorafenib failure and reported the predictors of progression-free survival, overall survival, post-progression survival, as well as the next-line treatments after regorafenib failure. We showed that some well-known survival predictors of sorafenib treatment and the response to prior sorafenib also had a prognostic role in patients with HCC undergoing regorafenib treatment. Preserved liver function and subsequent systemic therapy play important roles in survival after regorafenib failure. We conclude that the survival outcomes of regorafenib for HCC have improved in the era of multi-line sequential therapy. Preserved liver function and next-line therapy are important prognostic factors after regorafenib failure.

**Abstract:**

The predictors of response and survival in patients with hepatocellular carcinoma (HCC) receiving regorafenib remain unclear. This study aimed to delineate the determinants of response and survival after regorafenib and evaluate post-progression treatment and outcomes. We retrospectively enrolled 108 patients with unresectable HCC receiving regorafenib after sorafenib failure. Progression-free survival (PFS), overall survival (OS), post-progression survival (PPS) and post-progression treatments were evaluated. The median PFS, OS and PPS were 3.1, 13.1 and 10.3 months, respectively. Achieving disease control by prior sorafenib, early AFP reduction and hand-foot skin reaction (HFSR) were associated with significantly better radiologic responses. By multivariate analysis, the time to progression on prior sorafenib, HFSR and early AFP reduction were associated with PFS; ALBI grade, portal vein invasion, HFSR and early AFP reduction were associated with OS. ALBI grade at disease progression, main portal vein invasion, high tumor burden and next-line therapy were associated with PPS. The median PPS was 12 months in patients who received next-line therapy, and the PPS was comparable between patients who received next-line targeted agents and immunotherapy. In conclusion, survival outcomes of regorafenib for HCC have improved in the era of multi-line sequential therapy. Preserved liver function and next-line therapy are important prognostic factors after regorafenib failure.

## 1. Introduction

Hepatocellular carcinoma (HCC) is the sixth most common cancer in the world and the fourth leading cause of cancer-related mortality [1,2]. Systemic therapy is recommended as the standard of care for patients with HCC at advanced stages or patients with unresectable HCC who are unsuitable for loco-regional therapy (LRT), and it is estimated that about half of patients with HCC may receive systemic therapies at some time point during the course of HCC treatment [3]. For patients with unresectable HCC, the multi-targeted tyrosine kinase inhibitor (TKI) sorafenib has been the standard of treatment since 2008 [4,5], while regorafenib is the first drug approved as the second-line treatment after sorafenib failure for HCC. In the RESORCE trial, regorafenib significantly improved overall survival (OS) and progression-free survival (PFS) compared to the placebo [6]. Currently, the predictors of response and survival under regorafenib treatment for HCC have not been fully clarified. Regorafenib is structurally similar to sorafenib but appears to be more pharmacologically potent than sorafenib [7]. Therefore, regorafenib and sorafenib might share some common predictors of response and survival. Recent studies suggest that response to prior sorafenib treatment is associated with the outcomes of regorafenib treatment [8,9]. Several prognostic predictors in patients with HCC receiving sorafenib, such as the presence of hand-foot skin reaction (HFSR) [10], ALBI grade [11], early AFP response [12], progression pattern [13,14] and the PROSASH-II model [15], may also have prognostic value for regorafenib treatment.

With the advance of systemic therapies for HCC in the past decade, lenvatinib and subsequently the immunotherapy combinations of atezolizumab plus bevacizumab have been approved as first-line systemic therapies for HCC, whereas cabozantinib, ramucirumab, and immune checkpoint inhibitors (ICIs) pembrolizumab and nivolumab plus ipilimumab are also currently available second-line treatment options for HCC [16]. With the increased options for multiple lines of systemic therapies for HCC, the survival of patients with advanced HCC may improve over time. Several real-world studies of regorafenib for HCC reported that the OS might be longer than 12 months [9,17,18,19], suggesting that the OS of HCC grossly improves under multiple lines of sequential therapy. Nevertheless, the optimal subsequent treatment and the determinants of survival after sorafenib–regorafenib failure remain unclear. The aim of this study was to delineate the determinants of response and survival after regorafenib treatment and evaluate the post-progression outcomes in the era of multiple-line sequential systemic therapy.

## 2. Patients and Methods 

### 2.1. Patients

From May 2019 to September 2020, we retrospectively screened 115 patients with unresectable HCC in Taipei Veterans General Hospital who received regorafenib due to sorafenib failure. Patients were enrolled if they had histologically confirmed HCC or clinically confirmed HCC based on magnetic resonance imaging (MRI) or contrast-enhanced computed tomography (CECT) according to the diagnostic criteria of the American Association for the Study of Liver Diseases (AASLD) treatment guidelines [20]; patients with HCC were classified as being in Barcelona Clinic Liver Cancer (BCLC) stage C or in BCLC stage B and not suitable for trans-arterial chemoembolization (TACE) or other LRT. Patients were excluded if they were lost to follow-up within 2 months of treatment (*n* = 6) or had no measurable lesion when starting regorafenib (*n* = 1). For each cycle, the standard dose of regorafenib was 160 mg once daily for 3 weeks, followed by 1 week off therapy. Modification of the initial dose of regorafenib was allowed according to the presence of adverse events during prior sorafenib treatment. Regorafenib treatment was stopped when there was confirmation of disease progression by image studies or when patients experienced intolerable toxicity.

This study was approved by the Institutional Review Board in Taipei Veterans General Hospital (IRB number: 2021-04-006BC) and adhered to the guidance of the Declaration of Helsinki. The Institutional Review Board waived the need for written informed consent due to the retrospective nature of this study.

### 2.2. Patient Evaluation

Demographic profiles, biochemistry data and tumor characteristics at baseline and at the time of disease progression were recorded. The data included age, gender, duration and response to prior sorafenib treatment, prior or concurrent immune checkpoint inhibitors (ICI) therapy, concurrent loco-regional therapy (LRT), tumor size, tumor number, macrovascular invasion, extrahepatic metastasis, serum alpha-fetoprotein (AFP), platelet count, as well as levels of albumin, total bilirubin, creatinine, alanine aminotransferase (ALT), aspartate aminotransferase (AST), hepatitis B surface antigen (HBsAg) and anti-hepatitis C virus antibodies. The ALBI score and grade were calculated as previously described [21]. High tumor burden was defined as the presence of main portal vein thrombosis (Vp4), bile duct invasion or tumor involvement >50% liver volume [22]. The Prediction Of Survival in Advanced Sorafenib-treated HCC (PROSASH)-II model was calculated as previously described [15].

### 2.3. Outcome Assessment

Radiologic responses according to the Response Evaluation Criteria in Solid Tumors version 1.1 (RECIST v1.1) were evaluated every 8–12 weeks during treatment [23]. The objective response rate (ORR) was defined as the percentage of patients with a complete response (CR) or partial response (PR). The disease control rate (DCR) was defined as the percentage of patients with CR, PR or stable disease (SD). 

Progression-free survival (PFS) was defined as the time interval between the day of starting regorafenib treatment and the onset of progressive disease (PD). Overall survival (OS) was defined as the time interval between the day of starting treatment and death. Post-progression survival (PPS) was defined as the time interval between the day of PD and death. The tumor progression pattern was classified into intrahepatic or extrahepatic tumor growth (>20% increase in tumor size of the viable target lesions), new intrahepatic lesions, and new extrahepatic lesions (including new vascular invasion and/or metastasis) [13,14]. Early AFP response was defined as greater than a 10% reduction in AFP levels from baseline within 1 month of treatment [24].

### 2.4. Statistical Analysis

All statistical analyses were performed using IBM SPSS Statistics for Windows, Version 22 (IBM, Armonk, NY, USA). Values were expressed as mean ± SD or as median (range) when appropriate. We used the Mann–Whitney U test to compare continuous variables and the Pearson chi-square analysis to compare categorical variables. We used the Kaplan–Meier method to estimate survival rates and the log-rank test to compare survival curves between patient groups. We used the Cox proportional hazards model to analyze prognostic factors for survival. Variables that achieved statistical significance (*p* < 0.05) or those close to significance (*p* < 0.1) by univariate analysis were subsequently included in the multivariate analysis. Statistical significance was considered as a *p*-value < 0.05 determined by two-tailed tests. 

## 3. Results

### 3.1. Patient Characteristics

A total of 108 patients receiving regorafenib for unresectable HCC due to sorafenib failure were ultimately enrolled for analysis. The baseline characteristics of the 108 patients are summarized in Table 1. The majority of patients belonged to BCLC stage C (81.5%), Child–Pugh class A (84.3%), and 38 (35.2%) patients presented with a high tumor burden. Regorafenib was given as the second- and third- to fifth-line therapy after sorafenib failure in 88 (81.5%) and 20 (18.5%) patients, respectively. The median duration of prior sorafenib therapy was 3.9 months, and 59.1% and 51% of patients experienced dose reductions and hand-foot skin reactions (HFSR) during sorafenib treatment, respectively. Nineteen patients (17.6%) experienced prior ICI therapy, while sixteen (14.8%) and nineteen (17.6%) patients received concurrent LRT (TACE 14, radiofrequency ablation 2) and ICI therapy (nivolumab 10, pembrolizumab 3, atezolizumab 1, durvalumab 5), respectively. Sixty-two patients (57.4%) experienced dose reduction of regorafenib, and the most frequently reported adverse events were HFSR (29.6%), diarrhea (15.7%) and hypertension (23.1%).

### 3.2. Radiologic Response

Evaluations of the best radiologic response by RECIST v1.1 to regorafenib and to prior sorafenib treatment were available in 103 (95.4%) and 98 (90.7%) of all patients, respectively (Table 2). The ORR and DCR to regorafenib treatment in all patients were 10.7% and 43.7%, respectively. Three patients (2.9%), all in the second-line setting, achieved a complete response. The ORR and DCR to prior sorafenib treatment were 21.4% and 44.9%, respectively. In patients achieving disease control by prior sorafenib treatment, the DCR to regorafenib was significantly higher (59.1% vs. 29.6%, *p* = 0.006). Patients with HFSR and early AFP responses had significantly better radiologic responses. Patients with early AFP responses also had significantly higher ORR (21.4% vs. 0%, *p* = 0.004) and DCR (64.3% vs. 17.9%, *p* < 0.001). The ORR and DCR in patients who received regorafenib monotherapy were 8.6 and 39.1, respectively (Table S1). There was no significant difference in ORR and DCR between patients who did or did not receive concurrent LRT or ICI therapy (Table S1).

### 3.3. Factors Associated with Progression-Free Survival (PFS)

**Figure 1:**
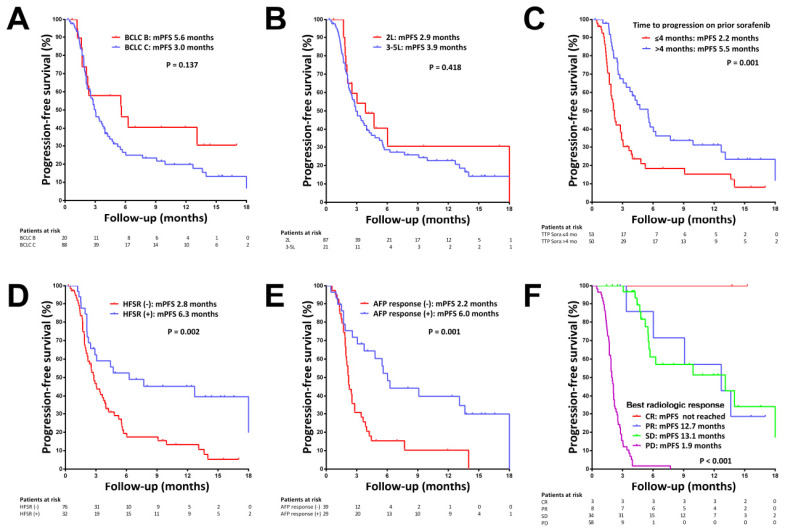
Kaplan–Meier curves of progression-free survival (PFS) in patients with HCC receiving regorafenib treatment. (**A**) PFS stratified by BCLC stage. (**B**) PFS stratified by lines of therapy. (**C**) PFS stratified by time-to-progression on prior sorafenib treatment. (**D**) PFS in patients with and without hand-foot skin reaction (HFSR). (**E**) PFS in patients with and without early AFP response. (**F**) PFS stratified by radiologic response by mRECIST criteria.

During a median follow-up period of 9.3 months, 78 (72.2%) patients developed disease progression with a median PFS of 3.1 months. The median PFSs were 5.6 and 3.0 months, respectively, in patients with BCLC stages B and C (*p* = 0.137, Figure 1A), and was 2.9 and 3.9 months in second-line and later-line settings, respectively (*p* = 0.418, Figure 1B). By multivariate analysis, TTP on prior sorafenib >4 months (hazard ratio (HR) = 0.563, *p* = 0.018, Figure 1C) was the only baseline predictor of PFS, while the presence of HFSR (HR = 0.238, *p* < 0.001, Figure 1D) and early AFP responses (HR = 0.397, *p* = 0.003, Figure 1E) were on-treatment predictors of PFS (Table 3 and Table S2). 

None of the three patients achieving CR had disease progression during the observation period, whereas the median PFSs in patients with PR and SD were 12.7 and 13.1 months, respectively (Figure 1F). We validated the PROSASH-II model for predicting RFS after regorafenib treatment, and a significantly poorer RFS was observed in PROSASH-II group 4 (*p* = 0.001, Figure S1A). 

### 3.4. Factors Associated with Overall Survival (OS)

**Figure 2:**
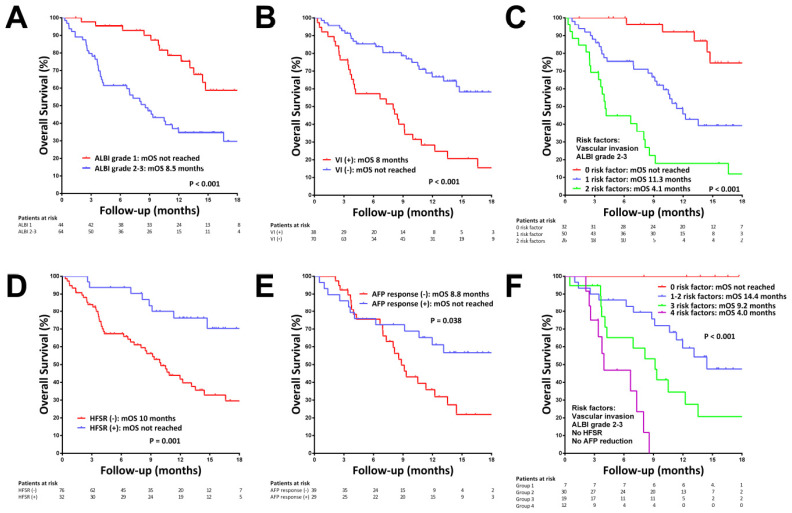
Kaplan–Meier curves for overall survival (OS) in patients with HCC receiving regorafenib treatment. (**A**) OS stratified by ALBI grade. (**B**) OS stratified by the status of portal vein invasion. (**C**) OS stratified by the number of baseline survival risk factors. (**D**) OS in patients with and without hand-foot skin reaction (HFSR). (**E**) OS in patients with and without early AFP response. (**F**) OS stratified by the number of baseline and on-treatment survival risk factors.

Fifty-two patients (48.1%) died during the observation period, with a median OS of 13.1 months. The median OSs in patients with BCLC stage C and second-line setting were 12 and 14.7 months, respectively (Figure S2A,B). The median OS was significantly better in patients with ALBI grade 1 (not reached vs. 8.5 months for ALBI grades 2–3, *p* < 0.001, Figure 2A) and Child–Pugh class A (14.7 vs. 4.1 months for Child–Pugh class B, *p* < 0.001, Figure S2C). By multivariate analysis, ALBI grades 2–3 (HR = 2.758, *p* = 0.002) and the presence of portal vein invasion (HR = 3.169, *p* < 0.001) were the baseline predictors of OS (Figure 2B). Combining the ALBI grades 2–3 and the presence of portal vein invasion could discriminate patients with high, intermediate and low risk of mortality (Figure 2C). The presence of HFSR (HR = 0.173, *p* < 0.001, Figure 2D) and early AFP response (HR = 0.450, *p* = 0.034, Figure 2E) were on-treatment predictors of OS (Table 3 and Table S3). Combining the risk factors of ALBI grade, portal vein invasion, HFSR and early AFP response could further stratify patients into four mortality risk groups (Figure 2F). The PROSASH-II model could also significantly stratify the OS after regorafenib treatment (median OS in groups 1, 2, 3, 4: not reached, 14.4, 8, 3.8 months, respectively; *p* < 0.001, Figure S1B).

### 3.5. Factors Associated with Post-Progression Survival (PPS)

Patient characteristics at disease progression and the tumor progression patterns for 78 patients with regorafenib failure are shown in Table 4. Twenty (25.6%) and 25 (32.1%) patients had deterioration of Child–Pugh class and ALBI grade at the time of disease progression, respectively.

**Figure 3:**
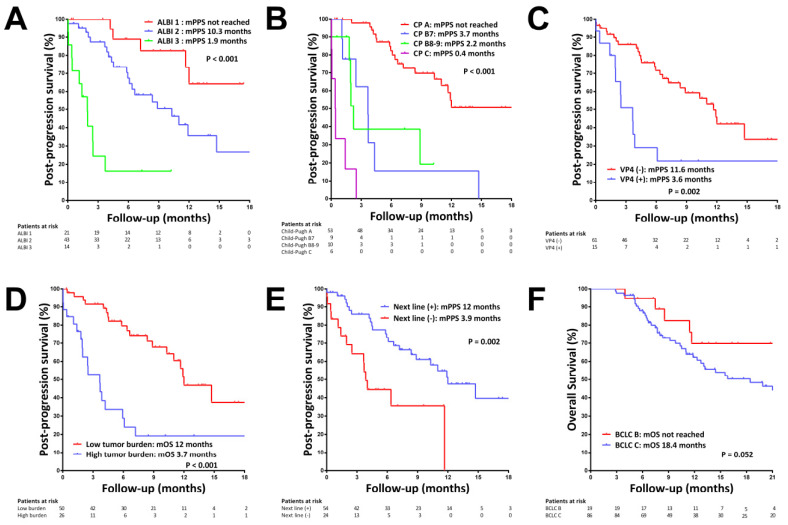
Kaplan–Meier curves for post-progression survival (PPS) after regorafenib failure and overall survival (OS) from starting sorafenib treatment. (**A**) PPS stratified by ALBI grade at disease progression. (**B**) PPS stratified by Child–Pugh class at disease progression. (**C**) PPS in patients with and without Vp4 vascular invasion. (**D**) PPS in patients with and without high tumor burden at disease progression. (**E**) PPS in patients who did and did not receive next-line therapy. (**F**) OS from starting sorafenib treatment stratified by BCLC stage.

The median PPS was 10.3 months. The median PPS in patients with ALBI grade 1 was not reached, and was 10.3 and 1.9 months in patients with ALBI grades 2 and 3, respectively (*p* < 0.001, Figure 3A). The median PPS in patients with Child–Pugh class A was not reached, and was 3.7, 2.2 and 0.4 months in patients with Child–Pugh classes B7, B8–9 and C, respectively (*p* < 0.001, Figure 3B). By multivariate analysis, ABLI grade (2 vs. 1: HR = 4.499, *p* = 0.006; 3 vs. 1: HR = 26.926, *p* < 0.001), the presence of main portal vein invasion (HR = 5.102, *p* = 0.007, Figure 3C), a high tumor burden (HR = 9.296, *p* < 0.001, Figure 3D) and receiving next-line therapy (HR = 0.369, *p* = 0.017, Figure 3E) were independent predictors of PPS (Table 3 and Table S4).

Fifty-four patients (69.2%) received next-line therapy after disease progression, including twenty-nine (53.7%) patients who received TKI monotherapy (levnatinib 22, cabozantinib 6, ramucirumab 1), thirteen (24.1%) who received ICI-based therapy (pembrolizumab plus lenvatinib 10, atezolizumab plus bevacizumab 2, nivolumab 1), seven (13%) who received TACE and five (9.3%) who received chemotherapy (FOLFOX: fluorouracil, leucovorin, oxaliplatin) (Table 4). The percentages of patients who received next-line therapies were 77.4%, 55.6%, 70% and 16.7% in patients with Child–Pugh classes A, B7, B8–9 and C, respectively (*p* = 0.009), and were 85.7%, 69.8% and 42.9% in patients with ALBI grades 1, 2 and 3, respectively (*p* = 0.009). The median PPS in patients who received next-line therapies was 12.0 months, and the individual median PPS by different next-line therapy is shown in Table 4. There was no significant difference in PPS among patients treated with next-line TKI or ICI-based therapy (*p* = 0.446).

### 3.6. OS since the Start of Prior Sorafenib 

The median OS from the start of sorafenib treatment was 21.2 months. The median OS was not reached in patients classified as BCLC B and was 18.4 months in patients classified as BCLC C (*p* = 0.052, Figure 3F). The median OS was not significantly different in the second-line and the third- to fifth-line settings (21.2 vs.24.4 months *p* = 0.982, Figure S2D).

## 4. Discussion

In this study, we reported the detailed survival outcomes of regorafenib for HCC in the era of multiple-line sequential systemic therapy. The ORR of 10.7% and the PFS of 3.1 months in this study were consistent with the results from RESORCE and recent real-world reports [6,8,9,17,18]. The DCR of 43.7% was lower than that in RESORCE but was similar to the largest real-world report from Korea [9]. The median OS in this study was 14.7 months in patients with Child–Pugh class A, which was longer than the data from RESORCE and previous real-world reports. The median OS of 4.1 months in patients with Child–Pugh B was also similar to the recent Korean report on regorafenib for patients with Child–Pugh B [25]. The median PPS of 10.3 months in our study suggests that post-progression treatment after sorafenib–regorafenib failure may further improve the OS in the era of multiple-line sequential treatment [26].

In our study, the TTP in prior sorafenib treatment was the baseline predictor of PFS under regorafenib treatment, which is consistent with the results of prior reports [8,9]. Although patients with a shorter TTP on prior sorafenib had a poorer tumor response and PFS with regorafenib, an exploratory study from RESORCE showed a consistent TTP benefit over placebo, irrespective of TTP on prior sorafenib, suggesting that shorter TTP on sorafenib does not preclude the survival benefit of regorafenib for HCC [27].

The presence of HFSR and early AFP reduction during regorafenib treatment were on-treatment predictors of radiologic response, PFS and OS. Recent studies showed that HFSR was not only a predictor of survival on sorafenib [10], but also a significant predictor for patients with HCC on regorafenib treatment [9,17]. Early AFP reduction has been shown to be an early predictor of response and survival to sorafenib and ICI therapy [12,24]. Our data showed that early AFP reduction also had a prognostic role for regorafenib treatment. 

Compatible with our findings, the ALBI score has been shown to be a predictor of HCC across the diverse BCLC stages, including patients who received sorafenib–regorafenib sequential therapy [11,28,29,30]. Several studies also reported that the presence of vascular invasion was a poor prognostic factor after sorafenib failure [31,32,33]. The PROSASH-II model, which comprised albumin, bilirubin, vascular invasion, extrahepatic spread, tumor size and AFP, has been shown to have good discriminative value in predicting the survival of patients with HCC receiving sorafenib treatment [15]. We also confirmed that the PROSASH-II model could discriminate PFS and OS in patients on regorafenib treatment. Based on the independent predictors of OS, we propose simple baseline and on-treatment risk scores that also have good discriminative value for predicting OS after regorafenib treatment. The risk scores could assist physicians with outcome prediction and considering an early switch to next-line treatment for patients with a high risk score.

The predictors of PPS and the impact of post-progression treatment after regorafenib failure remain unclear. In this study, the median PPS was 10.3 months, and 25% and 32% of patients showed a deterioration of Child–Pugh class and ALBI grade, respectively. Liver function reserve is an important determinant of PPS in this study, and patients with liver dysfunction at PD had less chance of receiving next-line therapy. In patients who maintained Child–Pugh A or ALBI grade 1, the median PPS was not reached during the observation period, whereas survival was significantly poorer in patients with liver function deterioration. Although progression patterns may have a prognostic impact after sorafenib failure [13,14], we did not observe a significant correlation between progression pattern and PPS after regorafenib failure, possibly due to the subsequent treatments after regorafenib failure. Next-line systemic therapy was shown to be an independent predictor of PPS after regorafenib failure, and the median PPS was 12 months in patients who were able to receive next-line therapy. The optimal third-line therapy after sorafenib–regorafenib failure remains unclear. Current guidelines and experts’ opinions suggest that other options for systemic agents could be applied as multiple-line sequential therapy [16,26,34]. In clinical practice, lenvatinib with or without ICI is the preferred subsequent systemic treatment after regorafenib, followed by cabozantinib. We did not observe a significant difference in PPS among patients treated with next-line TKI or ICI-based therapy. Although lenvatinib has only been evaluated in the first-line setting, recent real-world studies showed that lenvatinib could have survival benefits in the third-line setting after regorafenib failure [17,18,35]. In 2020, the phase Ib study of lenvatinib plus pembrolizumab showed promising results of high ORR and improved OS in the first-line setting [36], and this combination could also be a treatment option after sorafenib–regorafenib failure. Cabozantinib is the only systemic agent that has been investigated in the third-line setting in the CELESTIAL trial, and the survival benefit of cabozantinib is independent of the duration of prior sorafenib treatment [37]. Other treatment options, including ramucirumab, atezolizumab plus bevacizumab, and nivolumab, have also been applied as multiple-line sequential treatment options in real-world practice. In view of the PPS from our data according to different next-line systemic agents, lenvatinib or cabozantinib may be considered following sorafenib–regorafenib failure. In addition, lenvatinib plus ICI in combination with broadening modes of action might also be an option [38].

In the RESORCE trial, the median OSs from starting sorafenib were 26.0 and 21.5 months in the overall cohort and the Asian subgroup, respectively. Other real-world studies from Asia reported an OS of 25.3 to 28.5 months from starting sorafenib [9,17]. In this study, the median OSs from starting sorafenib were 28.3 and 13.1 months in patients with Child–Pugh classes A and B, respectively, and were 35.5 and 13 months in patients with ALBI grades 1 and 2, respectively. Consistent with previous studies, our data underline the crucial role of preserved liver function in the administration of multi-line sequential therapy and improved survival [39].

There are some limitations in this study. First, this is a retrospective study. Unintentional biases might exist in patient enrollment and the evaluation of clinical outcomes. Nevertheless, the National Health Insurance program in Taiwan enforced the strict regulation of clinical and image follow-up for the reimbursement of targeted therapies. Therefore, the majority of patients had regular clinical and image evaluations during sorafenib and regorafenib treatment for further drug reimbursement. Second, this is a single-center study from Taiwan, and the majority of patients had underlying HBV infections. Our findings need to be validated in other ethnicities and in HCC with other etiologies. Third, quality of life is an important issue during the application of systemic therapies for patients with HCC. However, quality of life measurements were not available in this retrospective study. Although TKI-related adverse events have adverse impacts on quality of life, patients with HFSR conferred better PFS and OS in our data. 

## 5. Conclusions

In conclusion, the survival outcomes of regorafenib for patients with HCC were consistent with those of the phase III trial result. Survival predictors and responses to sorafenib had a prognostic role in patients with HCC undergoing regorafenib treatment. Subsequent systemic therapy plays an important role in survival after regorafenib failure.

## Figures and Tables

**Table 1:** Characteristics of 108 patients receiving regorafenib therapy.

Variables	
Age (years)	65.3 ± 12.9
Male gender, *n* (%)	91 (84.3)
HCC etiology: HBV/HCV/HBV + HCV/Non-viral, *n* (%)	61/17/4/26 (56.5/15.7/3.7/24.1)
Lines of regorafenib therapy: 2/3/4/5, *n* (%)	88/12/6/2 (81.5/11.1/5.6/1.9)
Prior immune checkpoint inhibitors therapy, *n* (%)	19 (17.6)
Prior sorafenib duration (months) †	3.9 (0.5–44)
Dose reduction for sorafenib, *n* (%)	61 (59.8)
Hand-foot skin reaction during sorafenib treatment, *n* (%)	52 (51)
BCLC stage B/C, *n* (%)	20/88 (18.5/81.5)
Portal vein invasion, *n* (%)	38 (35.2)
Vp4	20 (18.5)
Extrahepatic metastasis, *n* (%)	71 (65.7)
Tumor size (cm)	4.65 ± 4.75
Multiple tumors, *n* (%)	74 (68.5)
High tumor burden, *n* (%) †	38 (35.2)
Child–Pugh class A/B, *n* (%)	91/17 (84.3/15.7)
ALBI grade 1/2/3, *n* (%)	44/63/1 (40.7/58.3/0.9)
Bilirubin (mg/dL)	0.99 ± 1.39
Albumin (g/dL)	3.74 ± 0.49
ALT (U/L)	49.5 ± 37.5
AST (U/L)	67.7 ± 58.6
Creatinine (mg/dL)	1.07 ± 0.88
Platelet (10^9^/L)	154 ± 96
AFP (ng/mL)	182.4 (1.2-1397041)
AFP > 400 ng/mL, *n* (%)	44 (40.7)
Follow-up period (months)	9.6 (0.3–29.0)
Initial dose of regorafenib: 160/120/80/40 mg	63/2/41/2 (58.3/1.9/38/1.9)
Dose reduction for regorafenib, *n* (%)	62 (57.4)
Adverse events during regorafenib, *n* (%)	
Hand-foot skin reaction	32 (29.6)
Diarrhea	17 (15.7)
Hypertension	25 (23.1)
Concurrent loco-regional therapy during regorafenib use, *n* (%)	16 (14.8)
Transarterial chemoembolization/radiofrequency ablation	14/2 (13/1.9)
Concurrent immune checkpoint inhibitors during regorafenib use, *n* (%)	19 (17.6)
Nivolumab/Pembrolizumab/Atezolizumab/Durvalumab	10/3/1/5 (9.3/2.8/0.9/4.6)
Disease progression, *n* (%)	78 (72.2%)
Death, *n* (%)	52 (48.1%)

† High tumor burden was defined as the presence of main portal vein thrombosis (Vp4), bile duct invasion or tumor involvement >50% liver volume. Sorafenib information was available for 102 (94.4%) patients.

**Table 2:** Best radiologic responses to regorafenib therapy by RECIST v1.1 criteria.

Radiologic Response †	CR	PR	SD	PD	ORR	DCR
Overall	3 (2.9%)	8 (7.8%)	34 (33%)	58 (56.3%)	11 (10.7%)	45 (43.7%)
Line of therapy						
2nd line (*n* = 83)	3 (3.6%)	6 (7.2%)	26 (31.3%)	48 (57.8%)	9 (10.8%)	35 (42.2%)
3rd–5th line (*n* = 20)	0 (0)	2 (10%)	8 (40%)	10 (50%)	2 (10%)	10 (50%)
*p* value				0.859	1.000	0.702
Achieving disease control by prior sorafenib						
Yes (*n* = 44)	1 (2.3%)	4 (9.1%)	21 (47.7%)	18 (40.9%)	5 (11.4%)	26 (59.1%)
No (*n* = 54)	1 (1.9%)	4 (7.4%)	11 (20.4%)	38 (70.4%)	5 (9.3%)	16 (29.6%)
*p* value				0.032	0.744	0.006
Presence of hand-foot skin reaction						
Yes (*n* = 32)	2 (6.3%)	3 (9.4%)	14 (43.8%)	13 (40.6%)	5 (15.6)	19 (59.4)
No (*n* = 71)	1 (1.4%)	5 (7.0%)	20 (28.2%)	45 (63.4%)	6 (8.5)	26 (36.6)
*p* value				0.032	0.310	0.052
Early AFP response						
Yes (*n* = 28)	2 (7.1%)	4 (14.3)	12 (42.9%)	10 (35.7%)	6 (21.4%)	18 (64.3)
No (*n* = 39)	0 (0%)	0 (0%)	7 (17.9%)	32 (82.1%)	0 (0%)	7 (17.9)
*p* value				<0.001	0.004	<0.001

† Evaluations of the best radiologic response to regorafenib and sorafenib treatment were available in 103 (95.4%) and 98 (90.7%) of all patients, respectively. CR, complete response; PR, partial response; SD, stable disease; PD, progressive disease; ORR, objective response rate; DCR, disease control rate.

**Table 3:** Independent factors associated with progression-free survival, overall survival and post-progression survival by multivariate analysis.

Variables		Multivariate	
		HR (95% CI)	*p*
Progression-free survival			
Baseline factor			
Time to progression on prior sorafenib (months)	>4/≤4	0.485 (0.302–0.781)	0.003
On-treatment factors			
Hand-foot skin reaction	Yes/No	0.238 (0.108–0.525)	<0.001
Early AFP reduction	>10%/≤10%	0.397 (0.214–0.737)	0.003
Overall survival			
Baseline factors			
ALBI grade	2-3/1	2.758 (1.458–5.216)	0.002
Portal vein invasion	Yes/No	3.169 (1.817–5.528)	<0.001
On-treatment factors			
Hand-foot skin reaction	Yes/No	0.173 (0.068–0.442)	<0.001
Early AFP reduction	>10%/≤10%	0.450 (0.215–0.940)	0.034
Post-progression survival			
Main portal vein invasion	Yes/No	5.102 (1.578–16.949)	0.007
High tumor burden	Yes/No	9.296 (3.379–25.578)	<0.001
ALBI grade	1	1	
	2	4.499 (1.541–13.137)	0.006
	3	26.926 (6.638–109.227)	<0.001
Next-line therapy	Yes/No	0.369 (0.163–0.838)	0.017

**Table 4:** Characteristics at disease progression in 78 patients with regorafenib failure.

Characteristics	Descriptive Analysis	Median Post-Progression Survival (Months)
BCLC stage B/C, *n* (%)	8/78 (10.3/89.7)	
Child–Pugh class A/B/C, *n* (%)	53/19/6 (67.9/24.4/7.7)	
Child–Pugh class deterioration, *n* (%)	20 (25.6)	
ALBI grade 1/2/3, *n* (%)	21/43/14 (26.9/55.1/17.9)	
ALBI grade deterioration, *n* (%)	25 (32.1)	
Bilirubin (mg/dL)	1.84 ± 2.25	
Albumin (g/dL)	3.43 ± 0.62	
ALT (U/L)	46.8 ± 49.0	
AST (U/L)	84.5 ± 119.6	
Creatinine (mg/dL)	1.11 ± 1.10	
AFP (ng/mL)	242 (1.39–823.19.9)	
AFP > 400 ng/mL, *n* (%)	34 (43.6)	
Tumor progression pattern		
Intrahepatic tumor growth	39 (50%)	
New intrahepatic lesions	33 (42.3%)	
Extrahepatic tumor growth	26 (33.3%)	
New extrahepatic lesions	24 (30.8%)	
Next-line therapy, *n* (%)	54 (69.2)	
Treatment types in 54 patients receiving next-line therapies		12.0
Child–Pugh class A at disease progression	41/53 (77.4%) *	Not reached
Child–Pugh class B7 at disease progression	5/9 (55.6%) *	4.3
Child–Pugh class B8–9 at disease progression	7/10 (70%) *	2.2
Child–Pugh class C at disease progression	1/6 (16.7%) *	0.3
ALBI grade 1 at disease progression	18/21 (85.7%) ^+^	Not reached
ALBI grade 2 at disease progression	30/43 (69.8%) ^+^	10.3
ALBI grade 3 at disease progression	6/14 (42.9%) ^+^	2.5
Tyrosine kinase inhibitor	29 (53.7%)	Not reached
Levnatinib	22 (40.7%)	Not reached
Cabozantinib	6 (11.1%)	Not reached
Ramucirumab	1 (1.9%)	No death event
Immune checkpoint inhibitor-based therapy	13 (24.1%)	11.9
Pembrolizumab + Lenvatinib	10 (18.5%)	8.9
Atezolizumab + Bevacizumab	2 (3.7%)	2.0 and 11.9
Nivolumab	1 (1.9%)	No death event
Transarterial chemoembolization	7 (13%)	Not reached
Chemotherapy (FOLFOX: fluorouracil, leucovorin, oxaliplatin)	5 (9.3%)	10.3

* *p* = 0.009; ^+^ *p* = 0.009.

## Data Availability

The data that support the findings of this study are available from the corresponding author upon reasonable request.

## Supplementary Materials

The following supporting information can be downloaded at: https://www.mdpi.com/article/10.3390/cancers14082014/s1, Figure S1: Kaplan–Meier curves for progression-free survival (PFS) and overall survival (OS) after regorafenib treatment. Figure S2: Kaplan–Meier curves for overall survival (OS) in patients with HCC receiving regorafenib treatment. Table S1: Best radiologic response to regorafenib therapy by RECIST v1.1 criteria. Table S2: Univariate and multivariate analyses of factors associated with progression-free survival. Table S3: Univariate and multivariate analyses of factors associated with overall survival. Table S4: Univariate and multivariate analyses of factors associated with post-progression survival.
