# Supplementary material for: Determinants of Survival and Post-Progression Outcomes by Sorafenib–Regorafenib Sequencing for Unresectable Hepatocellular Carcinoma
Source: Cancers (Basel). 2022 Apr 15;14(8):2014. doi: 10.3390/cancers14082014 (PMC9030368; doi:10.3390/cancers14082014)
Supplement: Supplementary file 1 [file cancers-14-02014-s001.zip › cancers-1656127-supplementary.pdf]

Table S1. Best radiologic response to regorafenib therapy by RECIST v1.1 criteria

Radiologic response*	CR	PR	SD	PD	ORR	DCR
Regorafenib monotherapy (n=69)	1 (1.4%)	5 (7.2%)	21 (30.4%)	42 (60.9%)	6 (8.6%)	27 (39.1%)
**Concurrent loco-regional therapy**						
Yes (n=16)	1 (6.2%)	0 (0%)	8 (50%)	7 (43.8%)	1 (6.2%)	9 (56.2%)
No (n=87)	2 (2.3%)	8 (9.2%)	26 (29.9%)	51 (58.6%)	10 (11.5%)	36 (41.4%)
P value				0.512	1.000	0.408
**Concurrent immune checkpoint inhibitors therapy**						
Yes (n=19)	1 (5.3%)	3 (15.8%)	5 (26.3%)	10 (52.6%)	4 (21.1%)	9 (47.4%)
No (n=84)	2 (2.4%)	5 (6%)	29 (34.5%)	48 (57.1%)	7 (8.3%)	36 (42.9%)
P value				0.299	0.116	0.919

*Evaluation of best radiologic response to regorafenib was available in 103 (95.4%) patients.

CR, complete response; PR, partial response; SD, stable disease; PD, progressive disease; ORR, objective response rate; DCR, disease control rate.

Table S2. Univariate and multivariate analyses of factors associated with progression-free survival

		Univariate		Multivariate Model I†		Multivariate Model II†	
		HR (95% CI)	P	HR (95% CI)	P	HR (95% CI)	P
**Baseline factors**							
Age	>60/≤60	0.906 (0.555-1.478)	0.693				
Sex	Male/ Female	0.850 (0.458-1.576)	0.606				
HBsAg-positive	Yes/No	1.599 (1.001-2.555)	0.049		NS		NS
Anti-HCV-positive	Yes/No	1.044 (0.594-1.837)	0.881				
Time to progression on prior sorafenib (months)	>4/≤4	0.475 (0.299-0.755)	0.002	0.485 (0.302-0.781)	0.003		NS
Achieving disease control by sorafenib	Yes/No	0.633 (0.396-1.012)	0.056		NS		NS

HFSR during sorafenib treatment	Yes/No	0.816 (0.519-1.285)	0.381		
Sorafenib dose reduction	Yes/No	1.289 (0.803-2.068)	0.293		
Second-line therapy	Yes/No	1.278 (0.702-2.326)	0.422		
BCLC stage	C/B	1.591 (0.856-2.957)	0.142		
Portal vein invasion	Yes/No	1.512 (0.939-2.435)	0.089	NS	NS
Vp4	Yes/No	1.822 (1.027-3.232)	0.040	NS	NS
Extrahepatic metastasis	Yes/No	1.067 (0.665-1.712)	0.788		
High tumor burden	Yes/No	1.223 (0.758-1.974)	0.409		
ALBI grade	2-3/1	1.561 (0.983-2.478)	0.059	NS	NS

AFP (ng/mL)	>20/≤20	1.448 (0.907-2.311)	0.120				
AFP (ng/mL)	>400/≤400	1.388 (0.884-2.178)	0.154				
ALT (U/L)	>40/≤40	1.327 (0.840-2.096)	0.225				
AST (U/L)	>40/≤40	1.472 (0.931-2.328)	0.098		NS		NS
Creatinine (mg/dL)	>1.2/≤1.2	0.609 (0.321-1.156)	0.130				
**On-treatment factors**							
Concurrent LRT	Yes/No	0.804 (0.433-1.493)	0.489				
Concurrent ICI	Yes/No	0.724 (0.396-1.323)	0.294				
Early AFP reduction	>10%/≤10%	0.368 (0.202-0.670)	0.001			0.397 (0.214-0.737)	0.003

HFSR during regorafenib treatment	Yes/No	0.433 (0.253-0.740)	0.002			0.238 (0.108-0.525)	<0.001
Regorafenib dose reduction	Yes/No	0.953 (0.607-1.496)	0.835				

Abbreviations: CI, confidence interval; HR, hazard ratio; NS, not significant; LRT, loco-regional therapy; ICI, immune checkpoint inhibitor; HFSR, hand-foot skin reaction.

†Model I included only baseline factors. Model II included both baseline and on-treatment factors.

Table S3. Univariate and multivariate analyses of factors associated with overall survival

		Univariate		Multivariate Model I†		Multivariate Model II†	
		HR (95% CI)	P	HR (95% CI)	P	HR (95% CI)	P
**Baseline factors**							
Age	>60/≤60	0.749 (0.426-1.318)	0.317				
Sex	Male/Female	0.703 (0.316-1.561)	0.386				
HBsAg-positive	Yes/No	1.023 (0.584-1.790)	0.937				
Anti-HCV-positive	Yes/No	1.515 (0.808-2.840)	0.195				
Time to progression on prior sorafenib (months)	>4/≤4	0.862 (0.497-1.495)	0.597				
Achieving disease control by sorafenib	Yes/No	1.008 (0.581-1.750)	0.977				

Second-line therapy	Yes/No	0.591 (0.315-1.111)	0.102				
BCLC stage	C/B	2.397 (0.953-6.030)	0.063				
Portal vein invasion	Yes/No	3.403 (1.960-5.910)	<0.001	3.169 (1.817-5.528)	<0.001		NS
Vp4	Yes/No	3.005 (1.636-5.521)	<0.001		NS	2.324 (1.063-5.080)	0.035
Extrahepatic metastasis	Yes/No	0.730 (0.417-1.281)	0.273				
High tumor burden	Yes/No	3.390 (1.945-5.910)	<0.001		NS		NS
ALBI grade	2-3/1	3.144 (1.672-5.910)	<0.001	2.758 (1.458-5.216)	0.002		NS
AFP (ng/mL)	>20/≤20	1.721 (0.953-3.108)	0.072				
AFP (ng/mL)	>400/≤400	1.370 (0.793-2.368)	0.259				

ALT (U/L)	>40/≤40	2.290 (1.318-3.980)	0.003		NS		NS
AST (U/L)	>40/≤40	2.707 (1.479-4.953)	0.001		NS	4.210 (1.726-10.266)	0.002
Creatinine (mg/dL)	>1.2/≤1.2	1.190 (0.596-2.376)	0.623				
**On-treatment factors**							
Concurrent LRT	Yes/No	0.439 (0.158-1.219)	0.114				
Concurrent ICI	Yes/No	0.718 (0.350-1.476)	0.368				
Early AFP reduction	>10%/≤10%	0.482 (0.239-0.973)	0.042			0.450 (0.215-0.940)	0.034
Hand-foot skin reaction	Yes/No	0.289 (0.135-03615)	0.001			0.173 (0.068-0.442)	<0.001

Abbreviations: CI, confidence interval; HR, hazard ratio; NS, not significant; LRT, loco-regional therapy; ICI, immune checkpoint inhibitor; HFSR, hand-foot skin reaction.

†Model I included only baseline factors. Model II included both baseline and on-treatment factors.

Table S4. Univariate and multivariate analyses of factors associated with post-progression survival

		Univariate		Multivariate	
		HR (95% CI)	P	HR (95% CI)	P
Age	>60/≤60	0.670 (0.347-1.294)	0.233		
Sex	Male/Female	0.695 (0.270-1.787)	0.450		
HBsAg-positive	Yes/No	0.899 (0.457-1.766)	0.757		
Anti-HCV-positive	Yes/No	1.647 (0.794-3.415)	0.180		
**Progression pattern**					
Intrahepatic size		0.791 (0.413-1.514)	0.478		
Intrahepatic number		1.107 (0.568-2.157)	0.765		
Extrahepatic size		0.709 (0.369-1.359)	0.300		
Extrahepatic number		1.080 (0.524-2.226)	0.835		
**Status at disease progression**					
BCLC stage	C/B	24.111 (0.239-2433.341)	0.176		
Portal vein invasion	Yes/No	3.106 (1.598-6.038)	0.001		NS
Vp4	Yes/No	2.937 (1.430-6.032)	0.003	5.102 (1.578-16.949)	0.007

Extrahepatic metastasis	Yes/No	1.094 (0.527-2.272)	0.809		
High tumor burden	Yes/No	3.780 (1.955-7.310)	<0.001	9.296 (3.379-25.578)	<0.001
ALBI grade	1	1			
	2	2.856	0.036	4.499 (1.541-13.137)	0.006
	3	13.935	<0.001	26.926 (6.638-109.227)	<0.001
AFP (ng/mL)	>20/≤20	2.977 (1.234-7.277)	0.015		NS
AFP (ng/mL)	>400/≤400	1.679 (0.877-3.214)	0.118		
ALT (U/L)	>40/≤40	1.795 (0.937-3.442)	0.078		NS
AST (U/L)	>40/≤40	2.309 (1.086-4.909)	0.030		NS
Creatinine (mg/dL)	>1.2/≤1.2	1.445 (0.653-3.198)	0.364		
Next-line therapy	Yes/No	0.339 (0.168-0.684)	0.003	0.369 (0.163-0.838)	0.017

Abbreviations: CI, confidence interval; HR, hazard ratio; NS, not significant.

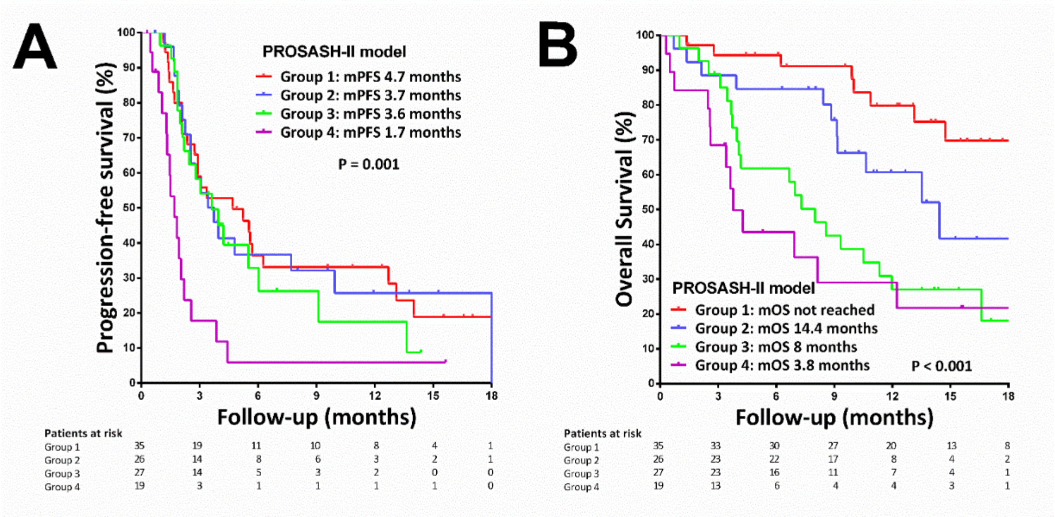

Figure S1. Kaplan-Meier curves of progression-free survival (PFS) and overall survival (OS) after regorafenib treatment. (A) RFS stratified by PROSASH-II model. (B) OS stratified by PROSASH-II model.

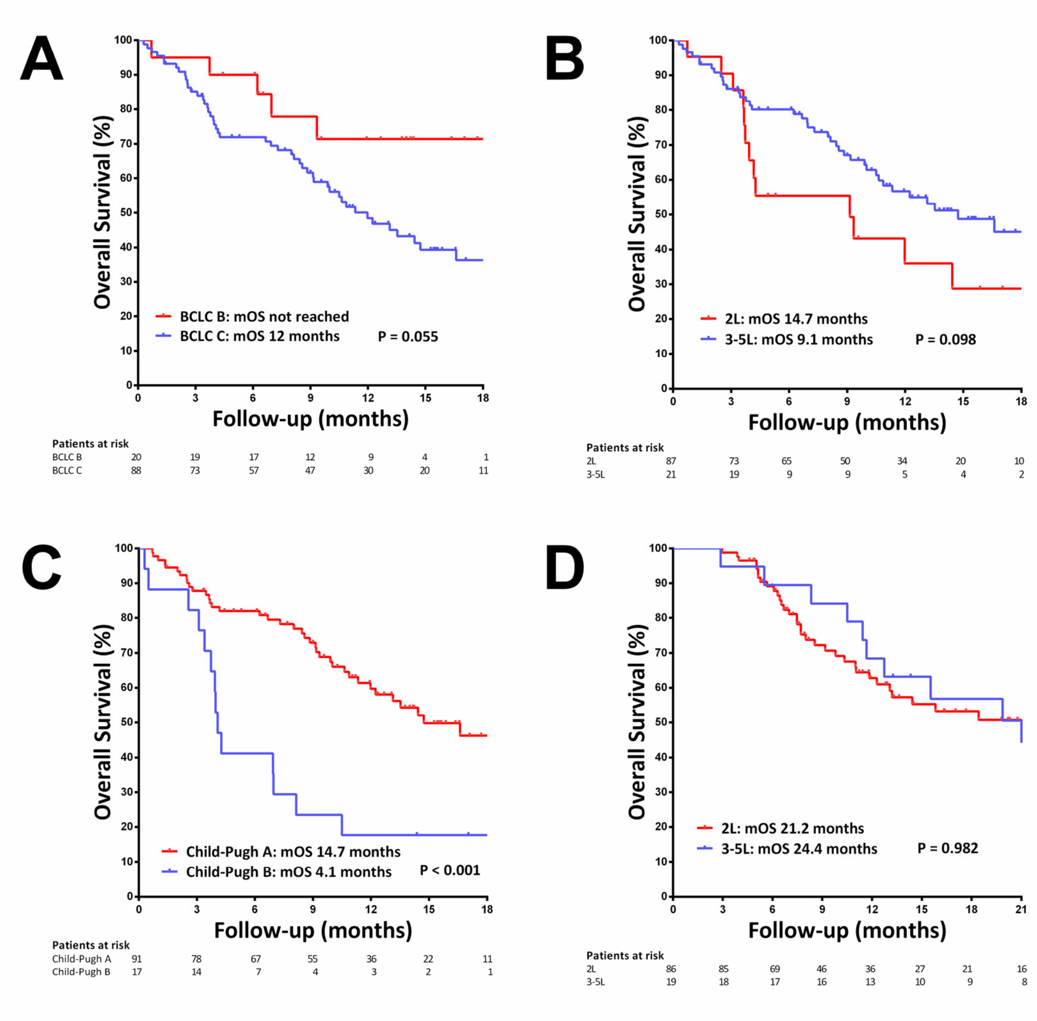

Figure S2. Kaplan-Meier curves of overall survival (OS) in HCC patients receiving regorafenib treatment. (A) OS stratified by BCLC stage. (B) OS stratified by lines of therapy. (C) OS stratified by Child-Pugh class. (D) OS from starting sorafenib treatment stratified by lines of therapy.
